# Down-Regulation of Cough during Exercise Is Less Frequent in Healthy Children than Adults. Role of the Development and/or Atopy?

**DOI:** 10.3389/fphys.2017.00304

**Published:** 2017-05-17

**Authors:** Silvia Demoulin-Alexikova, François Marchal, Claude Bonabel, Bruno Demoulin, Laurent Foucaud, Laurianne Coutier-Marie, Cyril E. Schweitzer, Iulia Ioan

**Affiliations:** ^1^EA 3450 DevAH - Laboratoire de Physiologie, Faculty of Medicine, University of LorraineVandœuvre-lès-Nancy, France; ^2^Service d'Explorations Fonctionnelles Pédiatriques, Hôpital d'enfants de Brabois - CHRU de NancyVandoeuvre-lès-Nancy, France

**Keywords:** cough, exercise, down-regulation, development, maturation, children, adult

## Abstract

Cough is typically associated with physical activity in children with asthma, but the characteristics of the relationship between cough and exercise has not been established under physiological conditions. The aim of the study was to describe the effect of exercise on the reflex cough response elicited by a single breath of capsaicin in non-asthmatic children. A group of non-asthmatic adults was studied as reference. Thirty children and 29 adults were recruited. The cough reflex sensitivity to capsaicin was first determined to establish the dose that provokes 5 cough efforts (C5). The number of coughs elicited by C5 (NC5) was then compared at baseline and during a standardized submaximal treadmill exercise. Data are expressed as median (interquartile range). Children and adults showed a significant decrease in NC5 (respectively from 5.0 (4.0–6.0) to 2.5 (2.0–4.0), *p* < 0.0005 and from 6.0 (5.0–7.0) to 2.0 (0.0–3.0, *p* < 0.0005). During exercise, NC5 was observed to decrease in all adult subjects, but in only 24/30 children (80%, *p* = 0.02). A trend for a higher incidence of personal and familial atopy was observed in children that lacked cough down-regulation during exercise compared with other children. It is concluded that the cough reflex response to capsaicin is down regulated by exercise in both children and adults. The effect however is less consistently observed in the former. The difference may reflect maturation of descending inhibitory pathways of the cough reflex, but may also be associated to atopy. The data stress the importance of assessing the time relationship of cough and exercise in questionnaire studies of asthma.

## Introduction

In children with respiratory disease, perception of dyspnoea may be difficult to estimate (Schweitzer and Marchal, 2009), wheeze may be missed, but cough appears at first hand easily identifiable from parental report. The symptom assessment however is subjective and may be biased by parental perception (Dales et al., 1997; Ioan et al., 2014). Diagnostic of the respiratory condition may benefit identification of a particular trigger such as exercise that is indeed sought by cough questionnaire studies (Suguikawa et al., 2009; Cichalewski et al., 2015; Boulet et al., 2017). Such formulations as “cough with exercise” (Langdeau et al., 2000), “cough during/after exercise” (Stelmach et al., 2016) or exercise-induced cough” (Nair et al., 2017) however do not establish whether cough occurs during or after the physical activity. As a first step toward identification of the relationship between cough and exercise in children with respiratory diseases, we aimed to characterize the ventilatory response to capsaicin during a brief run in healthy children.

Identifying the precise sequence has indeed potentially important implications, as the cough reflex has recently been shown to be down regulated at exercise in healthy adult humans (Lavorini et al., 2010). No data on the physiological relationship between cough and exercise are available in children. It is important to establish the characteristics of cough during exercise in children because the cough reflex changes during growth and important differences have been shown between children and adults (Chang, 2005, 2010). It is commonly accepted that cough is absent at birth and nociceptive stimulation of the airway fails to elicit a reflex cough response in babies (Thach, 2007) or neonatal animals (Marchal et al., 1982). Cough frequency and sensitivity to capsaicin challenge increase in childhood and around puberty and then decrease toward adulthood (Varechova et al., 2008). In addition, exposure to environmental pollution, such as tobacco smoke (ETS) and PM10 appears to have a significant impact on cough frequency and sensitivity during childhood (Demoulin-Alexikova et al., 2016). Thus, it appears important to determine whether cough is down-regulated during exercise in childhood, especially in view of the fact that children spend much time of their every-day life in physical activities.

The hypothesis is that the cough reflex is inhibited during exercise in healthy children. The main study objective was to determine whether the number of cough efforts elicited by a given concentration of capsaicin is decreased during near maximal treadmill exercise when compared to resting conditions. A group of healthy adult subjects served as reference for more precise assessment of effects of development.

## Materials and methods

### Study subjects

Healthy subjects were enrolled in the study based on following inclusion criteria: (a) medical history negative for asthma; (b) medical history negative for any chronic respiratory or systemic disease; (c) no history of cough lasting more than 2 weeks in the preceding 6 months; (d) no upper or lower respiratory tract infections for at least 15 days ahead of the test; (e) baseline ECG normal and (f) absence of obstructive pattern on spirometry. The exclusion criteria were as follows: (a) the birth before 34 week of gestational age; (b) the presence of cardiac malformation and/or heart rhythm disorder; (c) any medical contraindication for the practice of sport. Asthma was identified by one or more of the following during the past year: wheeze, cough or dyspnea at rest or on exertion and beneficial use of anti-asthma medications. Information about family history of atopy and a personal history of atopic disorders other than allergic asthma were obtained during careful history-taking. Written informed consents were obtained from the subject and/or parents, as necessary, and the study was approved by the local Ethics Committee (Comité de Protection des Personnes Est III).

### Study protocol

Body weight and height were measured; physical examination included auscultation of the chest and measurements of heart rate, transcutaneous oxygen saturation and arterial blood pressure. A 12 lead ECG and lung function tests were performed before capsaicin challenge and treadmill exercise.

The first step to reach study objective was to perform a cough provocation testing using capsaicin challenge in order to identify the concentration of capsaicin eliciting at least 5 cough bouts (C5). One hour after capsaicin challenge, the administration of C5 and saline aerosols in a random order was performed at rest (baseline). One hour thereafter, the dual random sequence C5–saline was administered during the period of near maximal exercise (exercise). The number of coughs evoked by C5 (NC5) at baseline or exercise was counted and served as the main outcome variable. The 1 h interval between successive challenges served to avoid the short-term effect of tachyphylaxis (Morice et al., 2007).

### Methods

#### Lung function

Spirometry was performed according to current guidelines (Miller et al., 2005) and Nitrous Oxide in exhaled breath (FE_*NO*_) measured by the single breath method using a NIOX-02-1000 Nitric Oxide Monitoring System (AEROCRINE AB, Solna, Sweden) (American Thoracic Society, European Respiratory Society, 2005).

#### Capsaicin inhalation cough challenge

The inhalation cough challenge using capsaicin as tussigenic agent was performed in agreement with the current ERS guidelines in adults (Morice et al., 2007), with adaptations for use in children (Varechova et al., 2008). Capsaicin was delivered using compressed air-driven nebuliser (model 646; DeVilbiss Health Care, Inc., Somerset, PA, USA) controlled by a dosimeter (KoKo DigiDoser-Spirometer; nSpire health Inc., Louisville, CO, USA) with an inspiratory flow regulator valve added (RIFR; nSpire health Inc., Louisville, CO, USA), to insure identical inspiratory flow and optimize reproducibility. A concentration—response curve was established using single breath delivery of doubling concentrations of capsaicin aerosol, from 0.61 to 1,250 μmol/l in children (Chang et al., 1996), and 0.49 to 1,000 μmol/l in adults (Morice et al., 2007). Normal saline was randomly aerosolized among test solutions, with successive aerosols being separated by a minimum 1 min interval. Each administration of saline and capsaicin aerosol was performed with the inhalation time set at 400 ms in children (Varechova et al., 2008) and 1,200 ms in adults (Morice et al., 2007). Number of coughs occurring within 15 s of capsaicin delivery was counted by 2 observers. Capsaicin challenge sessions were also video-taped, so as to check the initial counting whenever discordance would occur between observers. During capsaicin challenge, the concentration of capsaicin causing at least two coughs (C2) and C5 was retained for analysis. The end-point of cough challenge was the inhalation of C5.

#### Exercise

Exercise was performed on a motor-driven treadmill (h/p/cosmos mercury med 4.0, Nussdorf—Traunstein, Germany) in a climate room, where ambient temperature ranged 14–19°C and the water content of the inspired air was less than 10 mg/L (Anderson and Brannan, 2003). Heart rate was measured using a heart rate monitor (Polar B1, Helsinki, Finland). Treadmill speed and slope were adjusted during the first 2–3 min to increase heart rate to at least 80% of maximum value predicted for age. The near maximum exercise was pursued for another 3–4 min during which aerosols were tested. The connection to the respiratory apparatus was only necessary for the brief duration of the single breath inhalation.

### Statistics and data analysis

Statistical analysis was performed using SYSTAT 13 Package (San Jose, CA, USA). Normal distribution of data within each age group was tested using Kolmogorov-Smirnov test. Data are expressed as median (25th to 75th percentile) owing to non-normal distribution even after logarithmic transformation. NC5 was compared between baseline and exercise using Friedman test for repeated measurements. The effect of exercise was compared between children and adults from the percentage of change in NC5 during exercise from baseline using Mann-Whitney test. The remote possibility that normal saline would induce cough was accounted for by subtracting the corresponding number of coughs from that occurring after capsaicin (Chang et al., 1996). In what follows, the NC5 refers to the number of coughs evoked by C5 corrected for the effect of normal saline.

Between groups comparisons of quantitative data were performed using non parametric Mann-Whitney test. Between groups comparisons of qualitative data were performed using Chi-square test and Fisher exact test in the case of small sample size. Differences were considered significant at *p* < 0.05.

## Results

Thirty children (17 males) and 29 adults (12 males) were recruited. One adult female exhibited a sensation of retrosternal burning pain during the determination of C5 that recovered within hours but prevented completion of the study. The subject characteristics at baseline are detailed in Table 1.

**Table 1:** **Subject characteristics and spirometry at baseline**.

	**Children *N* = 30**	**Adults *N* = 28**
Age (years)	10.0 (8.0–11.0)	22.0 (20.0–24.0)
Height (cm)	140.0 (131.0–149.0)	170.0 (162.7–175.2)
Weight (kg)	33.0 (30.0–37.0)	64.0 (55.0–74.2)
Expired NO (ppb)	16.0^*^ (11.0–19.0)	19.5 (13.0–24.0)
SpO2 (%)	98.0 (98.0–98.0)	98.0 (97.0–99.0)
Heart rate (bpm)	90.0 (80.0–98.0)	70.0 (66.7–79.2)
FVC (% predicted)	104.0 (93.0–111.0)	100.5 (94.5–109.5)
FEV1 (% predicted)	103.5 (92.0–110.0)	98.0 (92.5–105.5)
Incidence of		
- passive/active smoking	4 (13.3)	2 (7.1)
- personal history of atopic disease	4 (13.3)	0 (0.0)
- other than asthma		
- family history of atopy	17 (56.7)	5^**^ (29.4)
- family history of asthma	6 (20.0)	2^**^ (11.7)

*Data are median (IQR)*.

* n = 22;

** *n = 17*.

The median capsaicin concentration that provoked at least 5 coughs during capsaicin challenge (C5) was 39.1 μmol/l (IQR: 9.8–156.3 μmol/l) in children and 23.4 μmol/l (IQR: 11.7–125.0 μmol/l; *p* = 0.4) in adults.

**Figure 1:**
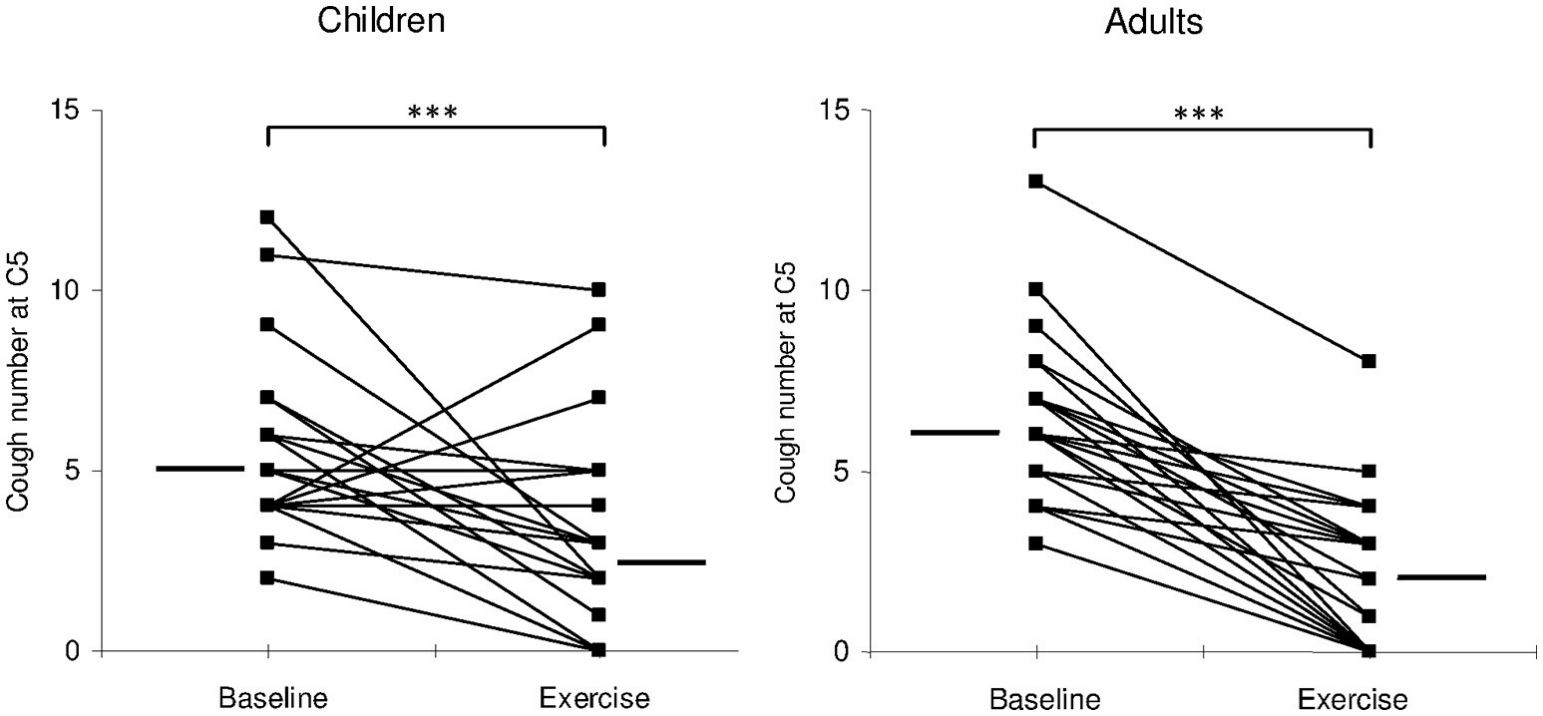
**Individual values (filed squares) and median values (thick lines) of cough number, provoked by C5 concentration of capsaicin, at rest (baseline) and during exercise (exercise)**. ^***^p < 0.0005.

NC5 was significantly reduced during exercise when compared to baseline, in both children (from 5.0 (4.0–6.0) to 2.5 (2.0–4.0); *p* < 0.0005) and adults [from 6.0 (5.0–7.0) to 2.0 (0.0–3.0); *p* < 0.0005; Figure 1]. The overall percent decrease in NC5 during exercise was not significantly different between the two groups [children: 60% (33–93%), *n* = 24 vs. adults: 71% (41–100%), *n* = 29; *p* = 0.3].

It is worth noting however that 6/30 children lacked NC5 reduction during exercise, while all adults that completed the study exhibited a decrease in NC5 (*p* = 0.02). Those 6 children where NC5 was not reduced during exercise had similar characteristics compared to the rest of the group (Table 2), except for a trend for a higher incidence of atopy in the personal history (33 vs. 8%) and in the family (83 vs. 42%). The difference however did not reach statistical significance.

**Table 2:** **Characteristics of children with and without cough down regulation during exercise**.

**Cough down regulation**	**No *n* = 6**	**Yes *n* = 24**	***p***
Males/females, *n*	4/2	13/11	ns
Age (years)	9.5 (9.0–11.0)	10.0 (8.0–12.2)	ns
Height (cm)	140.5 (139.0–148.0)	140.0 (130.5–149.5)	ns
Weight (kg)	34.0 (31.0–37.0)	33.0 (27.5–37.0)	ns
Expired NO (ppb)	10.5 (9.0–20.0)^**^	16.5 (14.0–19.0)^*^	ns
C2 (μmol/l)	5.2 (0.6–9.8)	4.9 (2.4–9.8)	ns
C5 (μmol/l)	97.7 (19.5–312.5)	39.1 (9.8–117.2)	ns
FVC (% predicted)	106.0 (97.0–109.0)	101.5 (92.0–113.0)	ns
FEV1 (% predicted)	108.5 (95.0–112.0)	102.5 (90.0–108.0)	ns
SpO_2_ (%)			
- at rest	98.0 (97.0–99.0)	98.0 (98.0–99.0)	ns
- 5th min after exercise	98.0 (97.0–99.0)	98.0 (98.0–99.0)	ns
Heart rate (bpm)			
- at rest	86.0 (74.0–97.0)	90.0 (80.0–100.0)	ns
- during exercise	168.25 (161.0–169.0)	165.0 (162.0–171.0)	ns
- 5 min after exercise	100.5 (90.0–120.0)	108.0 (99.0–111.0)	ns
Incidence of			
- passive smoking	0 (0.0)	4 (16.7)	ns
- personal history of atopic disease other than asthma	2 (33.3)	2 (8.3)	ns
- family history of atopy	5 (83.3)	10 (41.7)	ns
- family history of asthma	0 (0.0)	5 (20.8)	ns

*Data are median (IQR) or n (%). C2: capsaicin concentration that provoked at least 2 coughs, C5: capsaicin concentration that provoked at least 5 coughs. Between groups comparisons were performed using non parametric Mann-Whitney test for numeric variables and chi-square test (Fisher Exact test if n < 5) for categorical variables. ns, non-significant*.

* n = 18;

** *n = 4*.

## Discussion

To the best of our knowledge this is the first demonstration that the cough reflex response to a single breath capsaicin inhalation is down regulated during exercise in both healthy children and adults, with a similar magnitude of cough reduction in both groups. However, a small proportion of subjects lacked cough reduction during exercise which was exclusively found in children.

### Effect of exercise on cough

Exercise appears to modulate cough reflex response in a similar way as it does for the bronchomotor tone. Clinical studies in normal and asthmatic subjects and animal models of asthma have demonstrated airway dilation (Lee and Anderson, 1985; Cabral et al., 1999) and/or cough suppression (Lavorini et al., 2010; Tiotiu et al., 2017) during exercise while a transient airway narrowing (Lee and Anderson, 1985; Cabral et al., 1999; Anderson and Daviskas, 2000) and cough induction that occurs several minutes after exercise cessation is frequently seen in asthmatic subjects or athletes (Powell et al., 1996; Turmel et al., 2012; Bordeleau et al., 2014).

The hyperpnoea of exercise may cause drying of the airway, and subsequent increased hyperosmolarity of the airway surface lining fluid (Anderson et al., 1989; Boulet and Turcotte, 1991; Freed, 1995; Anderson and Daviskas, 2000; Anderson and Kippelen, 2008). There is increasing evidence that aerosol hyperosmolar solutions has the potential to provoke cough through activation of peripheral afferent nerve fibers not only in asthmatic but also in healthy humans and naïve animals (Pedersen et al., 1998; Koskela et al., 2004, 2005, 2008; Singapuri et al., 2008; Purokivi et al., 2011). The challenge of guinea pig airway with hyperosmolar solutions of NaCl (4%) or Mannitol (0.9 M) *in vitro* leads to activation of jugular ganglion neurons—projecting to trachea and main stem bronchi—that may trigger cough (Pedersen et al., 1998). Also, administration of Mannitol dry powder via an Osmohaler in humans causes cough in a dose dependent manner (Singapuri et al., 2008). However, when hyperosmolarity in airways is induced together with increased ventilation such as during dry air isocapnic voluntary hyperpnoea (Purokivi et al., 2011), the cough is elicited only rarely during the hyperpnoea in healthy subjects, and in asthmatics cough occurs mostly during the post-hyperventilation period. These findings, together with our results, suggest that inhibition of the reflex response to tussigenic agents during exercise is possibly due to modulation of sensory inputs at peripheral and/or central level.

A previous study in adults recently demonstrating the effect of exercise on cough used a quite different methodology where cough was induced by continuous nebulization of distilled water while the subject was exercising on a cyclo-ergometer (Lavorini et al., 2010). The convergent findings from both studies indicate that down regulation of the cough reflex should not depend on the nature of the afferent volley triggering cough, since nebulized distilled water activates mechanically sensitive “cough receptors” through low chloride content (Morice et al., 2007), while capsaicin selectively activates bronchial C-fibers through the opening of TRPV1 ion channels (Canning et al., 2014). The ventilatory response to exercise has strong potentials to modulate cough, as the voluntary isocapnic hyperventilation was observed to suppress cough as well as a similar level of exercise-induced hyperventilation in adults (Lavorini et al., 2010). Chest wall vibrations increase the cough threshold to citric acid (Kondo et al., 1998), a suggestion that hyperpnoea related increased input from mechanoreceptors such as those located to intercostal muscles and/or costo-vertebral joints inhibits the cough reflex (Javorka et al., 1994). Bronchodilation has also been documented in exercising humans (Freedman, 1992; Crimi et al., 2002) as well as in an experimental exercise animal model (Marchal et al., 2012), where the cough reflex was also found to be inhibited (Poussel et al., 2014). The decreased strain to the airway wall structure with bronchodilation may contribute to desensitize the airway cough receptor and therefore to down regulate the cough reflex (Smith et al., 1991). Indeed, numerous pre-clinical and clinical studies demonstrated antitussive properties of bronchodilators (β2-AR agonists) in different experimental settings and their antitussive activity was mostly attributed to the bronchodilation (Bolser et al., 1995; Lewis et al., 2007; Ohkura et al., 2010). However, recent findings show that β2-adrenoceptor agonists possess antitussive properties through direct inhibition of sensory nerve activity, independent of their bronchodilatory effect (Freund-Michel et al., 2010). Therefore, the exercise induced bronchodilation may not be the sole explanation to the observed cough attenuation during exercise. On the other hand, the increase in pulmonary blood flow activates pulmonary C-fibers located at the level of respiratory airways that are known to inhibit cough (Tatar et al., 1988) and in consequence the increased cardiac output associated with exercise is another possible contributor to the findings.

The cough reflex removes inhaled foreign particles and clears the lower respiratory tract from accumulated secretions, and thus assists breathing in order to adjust alveolar ventilation to metabolism and to maintain constant concentrations of oxygen and carbon dioxide in the blood (Canning, 2006). Those muscles that produce the periodic ventilatory activity are also involved in non-respiratory behaviors, and nociceptive reflexes result from the coordination of motoneuron networks controlled at brainstem, subcortical and cortical levels (Canning, 2006). Therefore, the behavioral drive to cough may ultimately compete with the metabolic drive to breathe. Several preclinical and clinical studies have shown that mechanically or chemically induced cough is down-regulated during hypoxic or hypercapnic stimulations (Nishino et al., 1989; Eckert et al., 2006).

Activation of cough afferents by airway irritation not only elicits cough motor act, but is responsible for the sensory information that undergoes subcortical (affective) and/or cortical (discriminative) processing generating cognitive cough sensations which have been defined as the urge-to-cough (Davenport and Hutchison, 2002; Davenport, 2009). Behavioral modulation either consciously suppress or reinforce the cough motor act (Davenport and Hutchison, 2002; Davenport, 2009; Davenport et al., 2009). It was suggested that urge to cough may allow activation of inhibitory pathways by higher brain centers in such situations ranging from social embarrassment to basic survival (Gracely et al., 2007). The presence of urge to cough during exercise may therefore contribute to suppress the nociceptive reflex, in order to favor the metabolic drive to breathe. This mechanism resembles central analgesia, where suppression of the pain reaction allows an organism to cope with the impending emergency (Amit and Galina, 1988). Indeed, Lavorini et al. (2010) have shown that cyclo-ergometer exercise was not associated with decreased urge to cough but only with decreased cough sensitivity (Lavorini et al., 2010). It appears therefore that the drive to breathe competes with—and overcomes—the drive to cough in situations where more ventilation is required by increased chemical drive or metabolism.

### Lack of cough down-regulation during exercise. role of atopy and/or development?

Altogether, cough down-regulation to tussigenic stimuli during exercise appears to act as important physiological adjustment increasing performance and appears to be disrupted under certain conditions. The mechanisms by which the physiologic adaptation can be altered has not been widely studied, however the association with airway inflammation is highly suggested (Hazucha et al., 1996). A recent demonstration of lack of cough down-regulation during electrical limb muscle stimulation in a rabbit model of late-phase response to allergen inhalation (Tiotiu et al., 2017) suggested a role for eosinophilic inflammation. A few studies conducted among high-level athletes showed a higher prevalence of cough during prolonged exercise or when performed under specific environmental conditions (Turmel et al., 2012; Boulet et al., 2017). In athletes, cough was mainly associated with asthma and exercise induced bronchoconstriction (Boulet et al., 2017), but neutrophilic inflammation caused by mild bronchial epithelial damage due to intense exercise may play a role, too (Yoshihara et al., 1995).

In the current study, the lack of cough down-regulation was found in healthy subjects with medical history negative for asthma and exhaled nitric oxide levels ≤20 ppb, suggesting absence of eosinophilic inflammation in the airways. The trend for a larger incidence of atopic disease other than asthma in the family and the personal history is worth mentioning. Increased capsaicin cough sensitivity was found in subjects suffering from allergic rhinitis (Pecova et al., 2005; Plevkova et al., 2006) or atopic dermatitis (Pecova et al., 2003) without any lower airway symptoms. On the other hand, little information is available on cough, cough sensitivity to tussigenic agents and food allergy. However, eosinophilic esophagitis may provoke isolated airway symptoms including cough even in the absence of any gastro-intestinal complaints (Kubik et al., 2016). Sensitization of cough reflex following application of sensory stimuli into the nose or esophagus are arguing for central mechanisms of cough sensitization from the esophagus and nose that are still poorly understood (Hennel et al., 2015). In the study of Turmel et al. (2012) cough during exercise was reported by 9–18% adult top athletes and 0–12% adult non-athlete subjects with highest prevalence during winter. The role of eosinophilic inflammation could be ruled out as all subjects had sputum eosinophils within the normal range (≤2%). On the other hand, cough during exercise might have been related to atopy, found in 67% of subjects. In our study, children with atopic disease other than asthma (*n* = 4) in the personal history had significant increase of C2 but not C5 as compared to non-atopic children. Moreover, NC5 in atopic children was not significantly reduced during exercise when compared to baseline (data not shown for clarity). These observations, together with no atopic disease in the personal history found in adults (all displaying cough down-regulation during exercise), strongly suggest the potential link between atopic diseases other than asthma and cough during exercise, however, further basic and clinical studies with confirmation in a larger group of subjects are required.

We are aware of little experimental evidence that would explain the less frequent cough down regulation by exercise in children compared to adults. As discussed above, atopy may play a role, however, excluding children with did not change our restults. Indeed, the cough down-regulation during exercise remained significantly less frequent in non-atopic children as compared to adults (*p* = 0.04; results not shown for clarity) suggesting the role of development. Numerous studies have already shown alterations in cough reflex characteristics throughout childhood and adolescence (Chang, 2005; Varechova et al., 2008; Ioan et al., 2014) owing to developmental changes in respiratory anatomy and physiology (Becklake and Kauffmann, 1999; Tulic et al., 2004), and evolving central processing of the nociceptive information (Walker, 2014). Recent advances in pain research have pointed toward the slow maturation of descending pathways inducing analgesia (Fitzgerald and Walker, 2009; Hathway et al., 2012) that may therefore be less efficient throughout childhood and early adolescence compared to adulthood (La Hausse de Lalouviere et al., 2014; Walker, 2014). The central processing of the afferent volley triggered by airway irritation may undergo similar developmental patterns of change resulting in less efficient suppression of cough in some children compared to adults. It is tempting to speculate that this phenomena might play a role in the increased incidence of tic (habit) cough, known to appear mostly in childhood and adolescence (Vertigan et al., 2015).

### Limitations of the study

The exercise protocol with single breath inhalation of capsaicin or saline aerosol developed for convenient use in children did not allow the continuous measurement of ventilation and CO_2_ that would indeed be optimal for the assessment of the level of ventilatory changes induced by exercise. However, since ventilation is tightly linked to heart rate during exercise (Treese et al., 1993) we thought that near maximal heart rate very likely attested near maximal ventilation during the step change. The protocol was also found to be easily performed by children with minimal constraint since the connection to the breathing apparatus was necessary only for a few breaths. The aural assessment did not allow a precise evaluation of the ventilatory response pattern that would otherwise have been helpful to differentiate cough from expiration reflex. On the other hand, the stimulus triggered in inspiration was much more likely to provoke a cough than an expiration reflex, based on existing experimental data (Poliacek et al., 2008; Varechova et al., 2010).

The exercise duration did not allow performing a complete capsaicin dose–response curve which has been reported to offer optimal reproducibility (Morice et al., 2007). However, the use of an inspiratory flow regulator valve warranted reproducible inspiratory efforts and consistent airway particle deposition and therefore allowed valid comparison of single breaths capsaicin before and after exercise. This test–retest methodology is otherwise widely employed in the experimentation of anti-tussive drugs (Venkatasamy et al., 2010; Cinelli et al., 2016; Mutolo et al., 2016). There is a possibility that the effect of long-term tachyphylaxis to capsaicin might partially account to a down-regulation of cough number between baseline and exercise, as we performed 2 single doses of C5 capsaicin concentration at the 60th and 120th min after complete capsaicin challenge. However, the highly significant NC5 difference between baseline and exercise should be considered mainly as the effect of cough down-regulation during exercise, as judging from the study of Morice et al. (1992) and the study of Matsumoto et al. (1994). In the first study, the authors did not found any significant difference in cough number elicited by the same dose of capsaicin between 240th and 360th min after capsaicin challenge (Morice et al., 1992). In the second one, no change in capsaicin threshold was found at 30th and 120th min when compared to the initial test (Matsumoto et al., 1994). However, no study has examined tachyphylaxis occurring between 60th and 120th min after capsaicin challenge, so its contribution to cough down-regulation during exercise in out set up cannot be definitely ruled out.

Given no available information in the literature about a possible link between cough during exercise and atopic disease other than asthma, we had no argument to determine serum total IgE levels or eosinophil counts in our subjects. This should indeed be done in the future basic and clinical studies aimed to better understand the underlying mechanisms of the lack of cough down-regulation during exercise.

## Conclusions

It is concluded that capsaicin induced cough is down-regulated during exercise in both children and adults. Peripheral mechanisms may be involved, such as desensitization of airway irritant receptors with exercise induced bronchodilation, increased rhythmic strain applied to the chest wall during hyperventilation, and increased pulmonary perfusion stimulating the pulmonary C-fibers. The modulation of cough may also take place at higher, subcortical and/or cortical levels through the activation of descending inhibitory pathway and these central mechanisms are likely candidates to explain the less frequent cough desensitization in children. However, more information is needed to clarify the mechanisms of cough down-regulation by exercise that may also be associated to atopy. An important practical implication from the current study is the necessity to identify the precise temporal relationship between cough and exercise in questionnaire studies of respiratory diseases, with more precise formulations than the “cough with exercise.”

## Ethics statement

This study was carried out in accordance with the recommendations of ANSM (Agence Nationale de Sécurité du Médicament et des Produits de Santé) and Ethics comitee CPP Est III with written informed consent from all subjects. All subjects gave written informed consent in accordance with the Declaration of Helsinki. The protocol was approved by ANSM and the Committees of Protection of Persons (Comités de Protection des Personnes) CPP Est III.

## Author contributions

SD, FM, and CS have prepared the project of this study. SD, FM, CS and CB managed preparatory phase of the study. SD and CB performed participant recruitment. SD, II, LC, CB, and BD performed lung function tests and capsaicin cough challenge measurements and assured technical assistance during exercise challenge. II, LF, SD, BD, and CB performed data collection and statistics. SD, II, and FM have prepared the draft of manuscript. SD, II, and FM completed the work and revised the final manuscript.

## Funding

This work was supported by Ministry of Higher Education and Research of France (Ministère de l'Enseignement supérieur et de la Recherche) under contract EA 3450 DevAH, by Nancy University Hospital Research programme under contract CPRC 2009-A00390-57 and by ARAIRLOR association.

### Conflict of interest statement

The authors declare that the research was conducted in the absence of any commercial or financial relationships that could be construed as a potential conflict of interest.
